# Population-specific, recent positive selection signatures in cultivated *Cucumis sativus* L. (cucumber)

**DOI:** 10.1093/g3journal/jkac119

**Published:** 2022-05-13

**Authors:** Xinrui Lin, Ning Zhang, Hongtao Song, Kui Lin, Erli Pang

**Affiliations:** MOE Key Laboratory for Biodiversity Science and Ecological Engineering and Beijing Key Laboratory of Gene Resource and Molecular Development, College of Life Sciences, Beijing Normal University, Beijing 100875, China; MOE Key Laboratory for Biodiversity Science and Ecological Engineering and Beijing Key Laboratory of Gene Resource and Molecular Development, College of Life Sciences, Beijing Normal University, Beijing 100875, China; MOE Key Laboratory for Biodiversity Science and Ecological Engineering and Beijing Key Laboratory of Gene Resource and Molecular Development, College of Life Sciences, Beijing Normal University, Beijing 100875, China; MOE Key Laboratory for Biodiversity Science and Ecological Engineering and Beijing Key Laboratory of Gene Resource and Molecular Development, College of Life Sciences, Beijing Normal University, Beijing 100875, China; MOE Key Laboratory for Biodiversity Science and Ecological Engineering and Beijing Key Laboratory of Gene Resource and Molecular Development, College of Life Sciences, Beijing Normal University, Beijing 100875, China

**Keywords:** *Cucumis sativus* L, cultivated cucumber, population-specific, recent positive selection, Fisher’s combination

## Abstract

Population-specific, positive selection promotes the diversity of populations and drives local adaptations in the population. However, little is known about population-specific, recent positive selection in the populations of cultivated cucumber (*Cucumis sativus* L.). Based on a genomic variation map of individuals worldwide, we implemented a Fisher’s combination method by combining 4 haplotype-based approaches: integrated haplotype score (iHS), number of segregating sites by length (nSL), cross-population extended haplotype homozygosity (XP-EHH), and Rsb. Overall, we detected 331, 2,147, and 3,772 population-specific, recent positive selective sites in the East Asian, Eurasian, and Xishuangbanna populations, respectively. Moreover, we found that these sites were related to processes for reproduction, response to abiotic and biotic stress, and regulation of developmental processes, indicating adaptations to their microenvironments. Meanwhile, the selective genes associated with traits of fruits were also observed, such as the gene related to the shorter fruit length in the Eurasian population and the gene controlling flesh thickness in the Xishuangbanna population. In addition, we noticed that soft sweeps were common in the East Asian and Xishuangbanna populations. Genes involved in hard or soft sweeps were related to developmental regulation and abiotic and biotic stress resistance. Our study offers a comprehensive candidate dataset of population-specific, selective signatures in cultivated cucumber populations. Our methods provide guidance for the analysis of population-specific, positive selection. These findings will help explore the biological mechanisms of adaptation and domestication of cucumber.

## Introduction

Locating population-specific, positive selection from genetic variation is a vital and considerably challenging task in evolutionary biology. Positive selection drives local adaptation (Casillas and Barbadilla 2017) and may also help clarify the mechanisms of biological evolution. Positively selected genomic regions were related to domestication (Li *et al.* 2016; Wang *et al.* 2016), life habits (Fumagalli *et al.* 2015), diseases (Wang *et al.* 2016), and specialization (Acer *et al.* 2019).

Living in a new environment, changing environmental factors (Acer *et al.* 2019) leads to a shift in the distribution of fitness effects for genomic variants (John and Seetharaman 2016). High-fitness selected variants spread rapidly and leave signatures in genomes. Research issues have utilized the signatures to detect positively selected genes that contribute to its adaption to the environments in *Arabidopsis thaliana* (Hancock *et al.* 2011) and rice (Liu *et al.* 2021). The signatures were also used to reveal domestication and breeding selection in crop (Wu *et al.* 2022).

Selection imprint signatures in genomes are used to detect targets that are selected, including a low genetic diversity, a shift in the site frequency spectrum of polymorphisms, and an excess of linkage disequilibrium (LD) (Stephan 2019). To capture the signatures, corresponding methods have been provided, including nucleotide diversity measures (Weir and Cockerham 1984; Chu and Wei 2020), site frequency spectrum-based approaches (Tajima 1989; Nielsen *et al.* 2005; Hua *et al.* 2010; Racimo 2016), and haplotype-based methods (Voight *et al.* 2006; Sabeti *et al.* 2007; Tang *et al.* 2007; Anna *et al.* 2014). Moreover, some of them are based on a single population, and others are based on the comparisons of multiple populations. However, research has reported that no single method is able to identify all of selective sweeps occurring at any given time (Hannah and Florian 2018); thus, combining several methods can greatly increase the power to pinpoint the selected region (Grossman *et al.* 2010; Alexandra *et al.* 2015).

Haplotype-based methods, as powerful tools to detect recent positive selection, have developed many relevant metrics, such as integrated haplotype score (iHS) (Voight *et al.* 2006), number of segregating sites by length (nSL) (Anna *et al.* 2014), cross-population extended haplotype homozygosity (XP-EHH) (Sabeti *et al.* 2007), and Rsb (Tang *et al.* 2007). IHS and nSL based on a single population perform well in selecting early stages (Alexandra *et al.* 2015), whereas XP-EHH and Rsb based on the comparison of 2 populations are powerful for beneficial alleles shortly before or at fixation (Sabeti *et al.* 2007; Tang *et al.* 2007; Alexandra *et al.* 2015). These methods have been widely used to reveal adaption and domestication of crops. XP-EHH detected genes associated with plant defense against insects and herbivores in tea (Zhang *et al.* 2021). In tomato, selective sweeps identified by iHS were related to fruit weight (Zhao *et al.* 2022). Signatures of soft sweeps underlying determinate growth habit were found in soya bean by haplotype-based methods (Zhong *et al.* 2017).

Cucumber (*Cucumis sativus* L.) is an important vegetable crop mainly cultivated in temperate and tropical regions of the world (Che and Zhang 2019). It is indigenous to India and the history of its domestication dates back to more than 2,500 years ago (Qi *et al.* 2013; Chomicki *et al.* 2020). A previous study generated a genomic variation map of 115 cucumber lines sampled from 3,342 accessions worldwide and divided the 115 lines into 3 cultivated groups and 1 wild group (Qi *et al.* 2013). In addition, common domestication sweeps were detected in the merged 3-in-1 cultivated group (Supplementary Fig. 1). Later, comparative analysis between wild and cultivated cucumbers was carried out using RNA-sequencing data (Abdel-Salam *et al.* 2020; Yan *et al.* 2020). Besides, 56 artificially selected cucumber inbred lines, where the genetic background was significantly different from the previous reported 115 lines, were assigned to group 1 and group 2, and selected regions were identified between group 1 and group 2 (Liu *et al.* 2019). Although there have been some studies on selection in cultivated cucumbers, none of them use haplotype-based methods, which are more powerful to recent positive selection. Thus, our research connecting genotypic variants and local adaption remains meaningful.

Here, we combined 4 haplotype-based approaches, which increased detection capabilities, and identified local adaptive signatures in each cultivated population. We attempted to address the specific selection in each cultivated population and furthermore investigated whether soft or hard sweeps are the common mode of adaptation in cultivated cucumber.

## Materials and methods

### Genomic variation

We downloaded the genomic variation from a previous study (Qi *et al.* 2013), which had sampled a core collection of 115 cucumber lines that captured a large proportion of the genetic diversity. Overall, the whole-genome resequencing of all 115 lines was performed with average coverage of 95.2% and depth of 18.3×. All these 115 lines were divided into 4 geographic groups: Indian, East Asian, Eurasian, and Xishuangbanna groups. The Indian group containing 30 lines mainly from India was identified as the wild group. The other 3 groups only contained cultivated lines: (i) East Asian group: 37 lines mainly came from China, Korea, and Japan; (ii) Eurasian group: 29 lines came from central and western Asia, Europe, and United States; and (iii) Xishuangbanna group: 19 landraces cultivated in the Xishuangbanna region of tropical southwestern China. Population structure and phylogenetic reconstruction supported that the 3 cultivated groups are monophyletic (Qi *et al.* 2013). The dataset encompassed 3,263,035 SNPs in chromosomes, and these SNPs were used for analysis.

To ensure the data quality, a series of quality filters were applied. We excluded individuals with more than 15% of missing data. Moreover, we filtered out singletons and SNPs with a proportion of missing data greater than 10% (Zhao *et al.* 2016). The process of quality control was applied using VCFtools v0.1.17 (Danecek *et al.* 2011). The remaining dataset (Supplementary Table 1) was used for downstream analysis.

### Identification of ancestral alleles

To calculate the values of iHS (Voight *et al.* 2006) and nSL (Anna *et al.* 2014), an ancestral state for each variant was necessary. We inferred the ancestral state for each variant utilizing principles of maximum parsimony (Langley *et al.* 2012).

First, we obtained 7-way, cucumber-based, pairwise alignments from the website http://cmb.bnu.edu.cn/cisRCNEs_cucurbitaceous/index.html, which contained 7 closely related species in Cucurbitaceae. Based on the phylogenetic tree of the 7 species from the website (Supplementary Fig. 2), the clade consisting of *C.sativus*, *Cucumis melo*, *Citrullus lanatus*, and *Lagenaria siceraria* was named the 4-way clade and the clade consisting of all 7 species was named the 7-way clade. Based on the phylogeny and whole genome alignments, we inferred the ancestral states. The ancestral allele state for each site in cucumber was determined if more than 2 species in the 4-way clade or 4 species in the 7-way clade were consistent with each other. In addition, the allele state in cucumber different from that in ancestors was set as “derived.” Sites with ancestral allele states that could not be determined by the above 2 clades were set as “missing.”

### Inference of haplotypes

To detect selection signatures based on phase data, the inference of haplotypes was carried out in BEAGLE 5.0 (Sharon and Brian 2007). Considering that a long sliding window may cause a high phasing error rate, for each population, we set the distance corresponding to half of the maximum mean *r*^2^ value as 1 block according to our analysis of the LD decay using PopLDdecay v3.40 (Chi *et al.* 2018).

### Detection of selection signatures within populations

Signals of selection within populations were evaluated by 2 methods, iHS (Voight *et al.* 2006) and nSL (Anna *et al.* 2014). IHS is a statistic comparing the extended haplotype homozygosity (EHH) (Sabeti *et al.* 2002) between derived and ancestral alleles within a population. NSL is a statistic measuring the length of a segment of homozygosity between derived and ancestral alleles in terms of the number of mutations in the remaining haplotypes in the dataset in the same region (Anna *et al.* 2014). The values of iHS and nSL were calculated and standardized using the rehh package (v3.2.1) (Gautier *et al.* 2017) and selscan v1.2.0a (Szpiech and Hernandez 2014) with default parameters, respectively. Phase data and ancestry status were required for both of the calculators, and the SNPs with “missing” ancestral allele states were ignored. Considering the frequency dependence of expected iHS and nSL values under neutrality (Tabangin *et al.* 2009), standardization was performed for markers binned with respect to the derived allele frequency at the focal marker. Since the standardized values of iHS and nSL approximately follow a standard Gaussian distribution (Voight *et al.* 2006; Anna *et al.* 2014), for each method for each SNP, a *P* value relative to the null-hypothesis of neutral evolution was assigned.

### Detection of selection signatures between populations

Signals of selection between the cultivated population and Indian population were evaluated using 2 methods, XP-EHH (Sabeti *et al.* 2007) and Rsb (Tang *et al.* 2007). XP-EHH compares the integrated EHH between populations. Rsb is a method that compares the lengths of haplotypes associated with the same allele between populations (Tang *et al.* 2007). The XP-EHH and Rsb values were calculated and standardized using the rehh package (v3.2.1) (Gautier *et al.* 2017). The formulas of XP-EHH and Rsb showed nondependence on ancestral status; therefore, for each cultivated-wild pair of populations, all polymorphic phased sites were considered. Since the standardized values of XP-EHH and Rsb follow an approximately standard Gaussian distribution as well (Sabeti *et al.* 2007; Tang *et al.* 2007), a similar *P* value was assigned for each SNP.

### Combining selection signatures

We used a Fisher’s combination method (Fisher 1954), which had been applied in human (Arciero *et al.* 2018), to combine the *P* values of the 4 methods used for detecting selection signatures. For each SNP, let *P_i_* be the *P* value of the test statistic using method *i*, (*i*∈{1, …, 4}). The Fisher’s combined test statistic is equal to:

$$T=-2\sum_{i=1}^{n}\ln\left(P_{i}\right)\;\left(n\in \left\{1,\ldots ,4\right\}\right)$$

which follows a chi-squared distribution with 2*n* degrees of freedom (Fisher 1954).

We adjusted the *P* value for multiple tests by applying Benjamini–Hochberg correction (Benjamini and Hochberg 1995). Sites with a false discovery rate less than 0.01 were considered selective sites.

### Functional annotation and enrichment analysis

We obtained genomic structural and functional annotation from CuGenDB (9930 v2) (Zheng *et al.* 2019). Gene ontology (GO) enrichment and pathway enrichment analysis were performed using the web tools in CuGenDB. Pfam domains annotation of protein-coding genes were predicted by PfamScan (El-Gebali *et al.* 2019) based on Pfam35.0.

### Classification of selection sweeps

To classify the selective sweeps into soft or hard sweeps, we performed simulations for the hard sweep and soft sweep scenarios using discoal (Kern and Schrider 2016), a coalescent simulator able to generate population samples conditioning on the fixation of an allele due to hard or soft sweeps. For each cultivated population, a 200-kb region was simulated based on its demographic history inferred by (Qi *et al.* 2013). A mutation rate of *μ* = 1 × 10^−7^ per generation (Qi *et al.* 2013) was used. Sweep scenarios were simulated with a positive additive selection coefficient *s* ∼ *U*[0,1], and the time of fixation looking backward in time *τ* ∼ *U*[0,0.005]. Soft sweeps were simulated as a mutation that arose neutral and turned beneficial at a frequency *e* ∼ *U*[0,0.2]. Hard sweeps were simulated from a *de novo* mutation that was never neutral, that is, *e *=* *0. The frequency at which the selection ended *f* ∼ *U*[*e*, 1]. We used 100,000 coalescent simulations for each scenario and a total of 600,000 simulations were performed.

We calculated Bayes factors by taking the ratio of distribution density of observed H12 and H2/H1 values in 2 types of simulated data (Garud *et al.* 2021). The python scripts for calculating H12 and H2/H1 values were provided on the website https://github.com/ngarud/SelectionHapStats (Garud *et al.* 2015). Bayes factors for each observed H12 and H2/H1 took the ratio of the support of hard and soft sweep scenarios. The support value was the number of data set generating H12 and H2/H1 values with a Euclidean distance <0.1 from the observed H12 and H2/H1 values. If the Bayes factor >1, we classified the selective site as a hard sweep. In contrast, if the Bayes factor <1, we classified it as a soft sweep. If the Bayes factor was 1 or the support value was 0 for both hard and soft sweep, we considered the selective sweep as an “unknown.”

## Results

### Signatures of adaptation in cultivated populations

To identify genomic signatures of selective sweeps in cultivated populations of cucumber living worldwide, we used 4 haplotype-based approaches: iHS (Voight *et al.* 2006), nSL (Anna *et al.* 2014), XP-EHH (Sabeti *et al.* 2007), and Rsb (Tang *et al.* 2007). IHS and nSL compare the lengths of haplotypes between derived and ancestral alleles in a single population, while XP-EHH and Rsb compare the lengths of haplotypes between a cultivated population and the wild population.

To infer haplotypes in each population, BEAGLE 5.0 (Sharon and Brian 2007) was applied. The slide window was determined by the *r*^2^ values used to measure LD decay. The *r*^2^ values were listed in Supplementary Table 2. The graph of LD decay in the 4 populations was shown in Supplementary Fig. 3. Finally, parameters in BEAGLE were obtained shown in Supplementary Table 3.

Filtered by the quality of SNPs and by the data requirements for each method, the genomic SNPs used in each method differed (Supplementary Table 4). For the iHS and nSL methods, the ancestral state for each variant was required; some SNPs were failed to determine the ancestral state, therefore, there were fewer SNPs than those used by XP-EHH and Rsb. For each SNP, the *P* value was calculated from each method detecting significant departures from the null hypothesis of neutral evolution (Voight *et al.* 2006; Sabeti *et al.* 2007; Tang *et al.* 2007). Using the above approaches, variants under selection with a *P* value threshold of 0.01 were detected in each cultivated population (Supplementary Table 4). More signatures of selection were identified by the methods based on 2 populations than by the methods based on a single population (Supplementary Figs. 4–6), which was mainly because of 2 causes: (i) more SNPs used by the methods comparing 2 populations and (ii) the significantly higher sensitivity of the methods based on 2 populations compared with the methods based on a single population (Supplementary Table 5). The overlaps of 4 approaches in 3 cultivated populations were similar (Supplementary Figs. 7–9). The intersection of XP-EHH and Rsb was the biggest, followed by the intersection of iHS and nSL. The overlapping sites of all 4 approaches were 38, 23, and 8 SNPs in East Asian, Eurasian, and Xishuangbanna population, respectively.

**Fig. 1.**
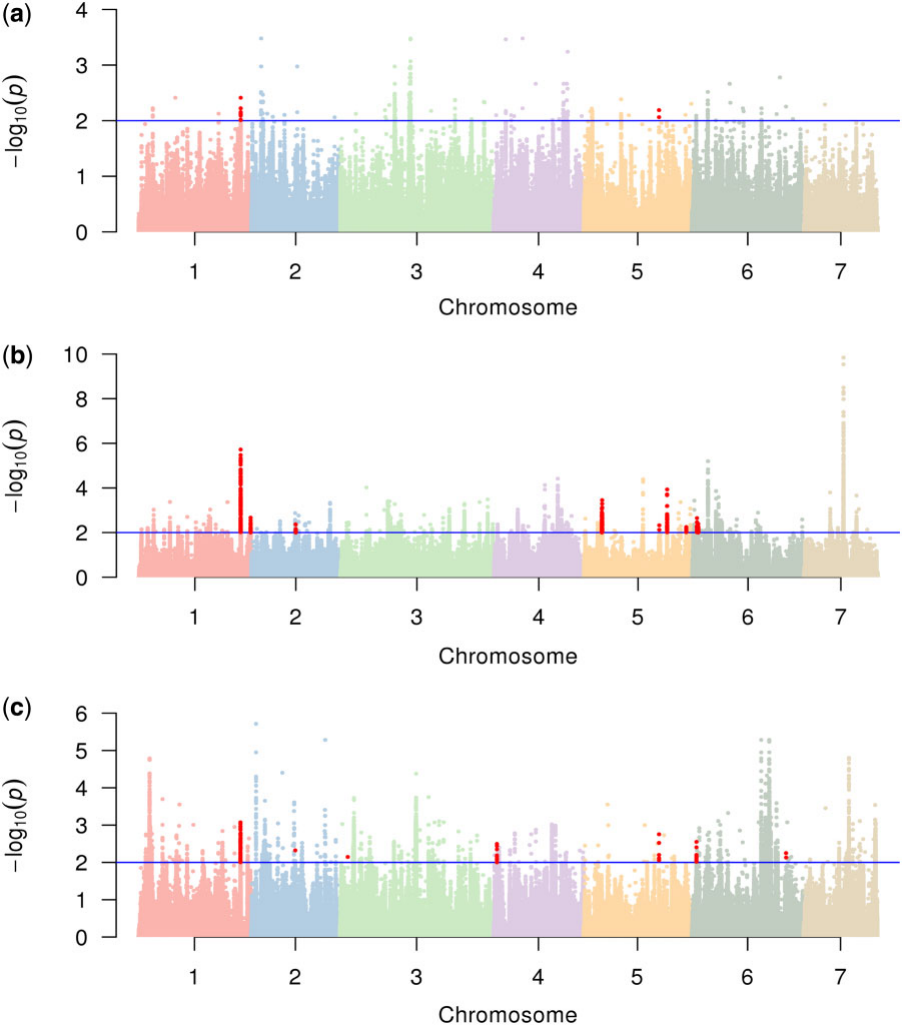
Manhattan plots of combined *P* values of local adaptation in the East Asian a), Eurasian b), and Xishuangbanna populations c). The blue horizontal line indicates the cutoff of the Benjamini–Hochberg-adjusted *P* value equal to 0.01, and the sites above are selective. The red dots represent selective variants located in domestication sweeps identified in a previous study (Qi *et al.* 2013). Manhattan plots were generated using the qqman package (Turner 2018).

Combining the *P* values from the 4 methods provides a concise way to integrate the above findings (Zaykin *et al.* 2007). *P* values were merged by Fisher’s combination, and candidate SNPs under positive selection were detected with a Benjamini–Hochberg-adjusted *P* value less than 0.01. According to the genomic structural annotation including 22,976 protein-coding genes in chromosomes, the SNPs under positive selection were further divided into 2 categories: in gene regions and in intergenic regions. In total, we identified 360 variants distributed in 22 genes in the East Asian population, in which 13 SNPs located in 2 previously identified domestication sweeps (Qi *et al.* 2013). Meanwhile, we found 2,248 variants located in 60 genes in the Eurasian population, in which 416 variants located in 9 selective regions detected by previous research (Qi *et al.* 2013). In addition, we detected 3,867 variants located in 120 genes in the Xishuangbanna population, in which 91 sites found in 7 domesticated regions in a previous study (Qi *et al.* 2013) (Table 1). In the 3 cultivated populations, most selected sites were located in the intergenic regions. The most selected sites were in the Xishuangbanna population. And the fewest selective sites were in the East Asian population (Table 1). This may be due to more genetic differentiation between Indian population and Xishuangbanna population than between Indian population and the others. The overlapping selective SNPs from a previous study were mainly located on chromosomes 1 and 5 in the 3 populations (Fig. 1).

**Table 1.** Variants showing the strongest signatures of positive selection in the 3 cultivated populations.

Population	SNPs^*a*^	Previously detected SNPs^*b*^	Gene regions^*c*^	Intergenic regions^*d*^	Genes^*e*^
East Asian	360	13	44	316	22
Eurasian	2,248	416	226	2,022	60
Xishuangbanna	3,867	91	812	3,055	120

a Number of selective SNPs with Benjamini–Hochberg-adjusted *P* value < 0.01.

b Number of selective SNPs detected by a previous study (Qi *et al.* 2013).

c Number of selective SNPs located in gene regions.

d Number of selective SNPs located in intergenic regions.

e Number of genes harboring selective SNPs under selection.

**Fig. 2.**
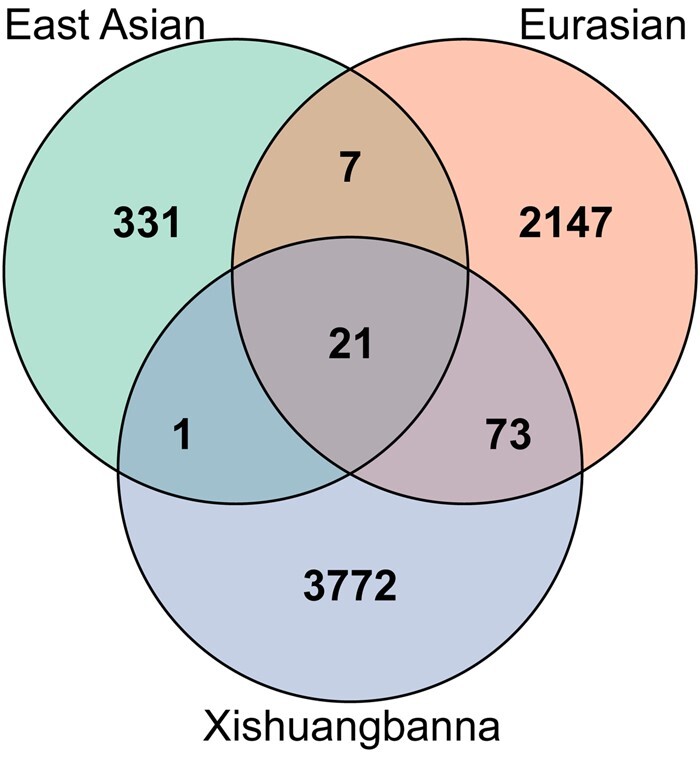
Venn diagram for selective sites in cultivated populations. Variants with Benjamini–Hochberg-adjusted *P* values less than 0.01 were identified as selective sites. A Venn diagram was generated using the package VennDiagram v1.6.20 (Chen and Boutros 2011).

To investigate the specific sites under selection in each cultivated population, we obtained the uniquely selective sites in each cultivated population. Most selective sites were population specific, consistent with the hypothesis of independent origin of the 3 cultivated populations (Qi *et al.* 2013; Chomicki *et al.* 2020). There were 331 SNPs under specific selection in the East Asian population, 2,147 variants in the Eurasian population and 3,772 sites in the Xishuangbanna population (Fig. 2). In the East Asian population, we found 7 SNPs leading to the change of amino acids and 72 SNPs localized at potential promoters of genes (defined as the 2-kb region upstream of the transcription start site) (Schwope *et al.* 2021). In Eurasian, 19 missense variants and 761 SNPs located in promoter regions were observed. In Xishuangbanna, 73 missense variants and 1,095 SNPs localized at gene promoters were observed. We also obtained 21 SNPs under selection shared by the 3 cultivated populations, which were all located in intergenic regions (8 of them localized at gene promoters).

We performed GO and pathway enrichment analysis to evaluate if there were any gene classes overrepresented. In the Xishuangbanna population, GO terms related to basic biological activities were observed (Table 2). In the East Asian population, a pathway related to tetrahydrofolate biosynthesis was enriched. In the Eurasian population, two pathway related to l-lysine biosynthesis was overrepresented. In the Xishuangbanna population, a pathway related to l-*N^δ^*- acetylornithine biosynthesis was enriched (Table 3).

**Table 2.** GO terms obtained by enrichment analysis in the Xishuangbanna population.

GO term	Description	Ontology^*a*^	Adjusted *P* value
GO: 1902903	Regulation of supramolecular fiber organization	BP	0.01147
GO: 0051493	Regulation of cytoskeleton organization	BP	0.02129
GO: 0003918	DNA topoisomerase type II (ATP-hydrolyzing) activity	MF	0.02981
GO: 0061505	DNA topoisomerase II activity	MF	0.02981
GO: 0016308	1-Phosphatidylinositol-4-phosphate 5-kinase activity	MF	0.04457
GO: 0019013	Viral nucleocapsid	CC	0.02420
GO: 0019028	Viral capsid	CC	0.02903
GO: 0019012	Virion	CC	0.03451
GO: 0044423	Virion part	CC	0.03451

a Ontology covers 3 domains: BP means biological process, MF means molecular function, and CC means cell component.

**Table 3.** Pathway enrichment analysis in the cultivated populations.

Population	Pathway ID	Pathway name	Adjusted *P* value
East Asian	PWY-6614	tetrahydrofolate biosynthesis	0.003
Eurasian	PWY-5097	l-Lysine biosynthesis VI	0.0285
Eurasian	PWY-724	Superpathway of l-lysine, l-threonine and l-methionine biosynthesis II	0.0330
Xishuangbanna	PWY-6922	l-*N^δ^*-acetylornithine biosynthesis	0.0229

### Unique selective signatures in the East Asian population

In the East Asian population, we found 44 specific selective sites located in 22 genes and 287 sites distributed in intergenic regions (Supplementary Table 6). In the 287 intergenic sites, 70 sites located in potential promoter regions. These genes were related to reproduction, plant response and resistance, and biosynthesis (Supplementary Table 7).

We found 2 genes related to reproduction, one of which is *WRKY27* gene (Csa3G354510) with 3 selective nonsynonymous variants, influencing pollen viability (Mukhtar *et al.* 2017). In addition, genes involved in plant response and resistance were detected, including the aquaporin (*AQP*) gene (Csa3G345890) which plays key roles in drought, flooding, nutrient availability, response to temperature and light conditions (Kaldenhoff and Fischer 2006), and a disease resistance response protein gene (Csa4G280640) in dirigent family with a selective synonymous variant. Moreover, we found some genes related to biosynthesis processes, such as the UDP-glycosyltransferase 1 (*UGT1*) gene (Csa3G608710) with a selective nonsynonymous variant, disrupting the biosynthesis of littorine and its tropane alkaloids derivatives (hyoscyamine and scopolamine) (Qiu *et al.* 2020). These findings hinted that there was existing selection of reproduction and disease resistance, and local adaption for light and temperature in the East Asian population.

### Unique selective signatures in the Eurasian population

In the Eurasian population, a total of 226 specifically selective sites were located in 60 genes, and 1,921 sites were located in intergenic regions (Supplementary Table 8). In the 1,921 intergenic sites, 743 sites located in potential promoter regions. We further found that the genes were involved in regulation of fruit length, development, response to environment, and DNA repair (Supplementary Table 9).

We found a gene Csa6G177440 located in the short-fruit 1 locus, which significantly decreased fruit length (Wang *et al.* 2017), and a gene, E3 ubiquitin-protein ligase *MARCH2* (Csa3G186720), that regulates plant development, including root development, organ size decisions, flowering time, and plant–environment interactions, such as drought and ABA (Shu and Yang 2017). Moreover, genes involved in the response to the environment were observed. For instance, the sesquiterpene synthase (*TST*) gene (Csa3G021130) defends against herbivores by their volatile terpenes (Liu *et al.* 2020), and the dehydration responsive element-binding transcription factor 1B (*DREB*) gene (Csa3G180260) enhances tolerance to freezing temperatures, drought, and high salinity (Agarwal *et al.* 2017). Furthermore, gene Csa3G912890 with 2 selective nonsynonymous variants and gene Csa5G424880 with a selective nonsynonymous variant were related to DNA repair. These findings suggested that the selective genes in the Eurasian population were related to the shorter fruit length, response to heat, drought and salt stress, pathogens and herbivore resistance.

### Unique selective signatures in the Xishuangbanna

In the Xishuangbanna population, we found 812 specifically selective sites located in 120 genes and 2,960 selective sites in the intergenic region (Supplementary Table 10). In the 2,960 intergenic sites, 960 sites located in potential promoter regions. The genes were involved in resisting stress and pathogens, regulating developmental processes, biosynthesis, and flesh thickness (Supplementary Table 11).

Genes related to resistance stress and pathogens were under selection. For example, the thaumatin-like protein (*TLP*) gene (Csa3G003990), containing a selective synonymous variant located in the Thaumatin domain, enhances drought and salt stress tolerance (de Jesus-Pires *et al.* 2020). The ABC transporter G family member (*ABCG*) gene (Csa6G434390) with 5 synonymous variants, of which 2 localized at ABC2 membrane family, is involved in ABA tolerance and plant resistance against *Pseudomonas syringae* pv. *tomato* DC3000 (*Pst* DC3000) in Arabidopsis (Ji *et al.* 2014). Genes involved in both stress resistance and regulating developmental processes were detected. For example, the *WRKY6* gene (Csa7G328830) with a selective nonsynonymous variant and a synonymous variant was detected serving functions in plant senescence processes, pathogen defence mechanisms, abiotic stress responses, and regulation of the accumulation of fatty acids during seed development (Song *et al.* 2020). In addition, genes regulating developmental processes were found, such as the mandelate racemase/muconate lactonizing protein (*DXS1*) gene (Csa2G404880) with a selective nonsynonymous variant located in the MR_MLE_C domain, which has been proposed serving fruit carotenoid biosynthesis and is required for the development and survival of tomato plants (Garcia-Alcazar *et al.* 2017), the DNA topoisomerase 1 (*TOP1*) gene (Csa3G063680), which modulates auxin-regulated root development in rice (Shafiq *et al.* 2017), and Csa2G058670 gene controlling flesh thickness (Xu *et al.* 2015). These results implied that local adaptation signatures in the Xishuangbanna population might be related to salt, temperature and bacterial pathogen resistance, root development, carotenoid biosynthesis, and thin fruit flesh.

### Soft selective signatures common in cultivated cucumbers

**Fig. 3.**
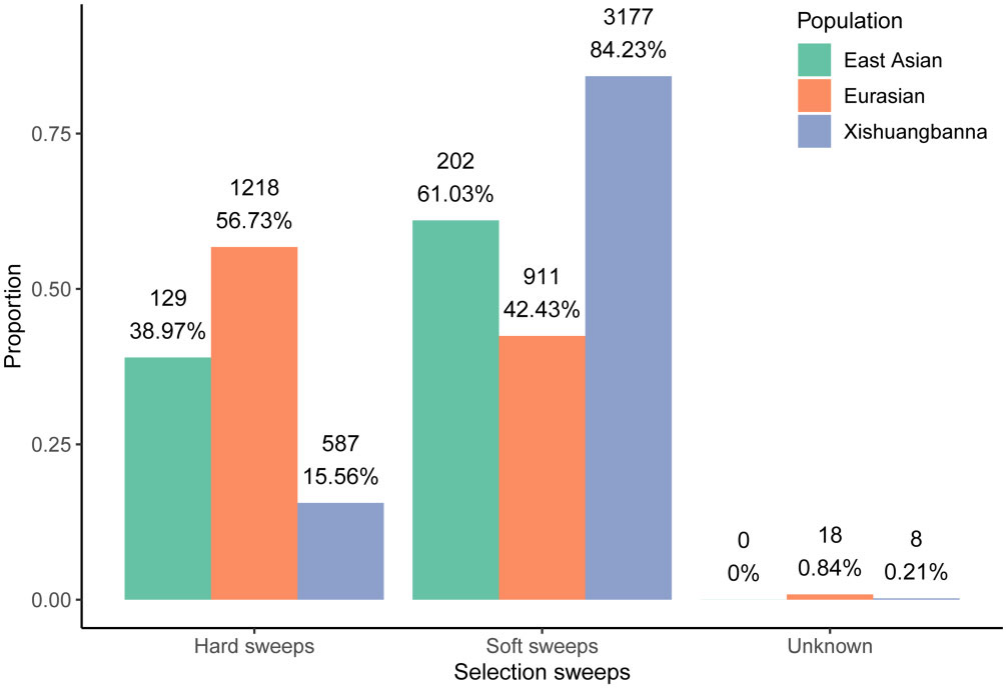
Proportion of selective sweeps in cultivated populations. In each bar, the count and proportion of specifically selective sites for each sweep corresponding to the population are indicated.

A selective sweep arises if a beneficial allele occurs at some recent time in the past and goes to fixation (Stephan 2019). This classic selective sweep model was defined as “hard sweep” formed by the *de novo* mutation (Hermisson and Pennings 2005). In contrast, multiple adaptive alleles sweep through the population at the same time was referred to as the “soft sweep” (Messer and Petrov 2013), which can help population rapidly respond to novel environmental challenges, rather than wait for a novel mutation to arise (Schrider and Kern 2017). Research has observed that soft sweeps are the dominant mode of adaptation in maize (Beissinger *et al.* 2014). Recent studies have reported that local adaptation and domestication indeed show signatures of such soft selective sweeps (Zhong *et al.* 2017; Stetter *et al.* 2020; Wu *et al.* 2022). Therefore, we further wanted to investigate whether soft sweeps contribute to adaptations in cucumber. We compared the signatures of hard sweeps with those of soft sweeps in the cultivated populations. Based on the Bayes factor (Garud *et al.* 2021), selective sites were divided into 3 groups: hard sweeps, soft sweeps and unknowns. We found that 61.03%, 42.43%, and 84.23% of sites are soft sweeps in the East Asian, Eurasian, and Xishuangbanna populations, respectively, which were significantly greater than those of hard sweeps except for Eurasian (Fig. 3). These results indicated that soft sweeps are the dominant mode of adaptation in the East Asian and Xishuangbanna.

**Fig. 4.**
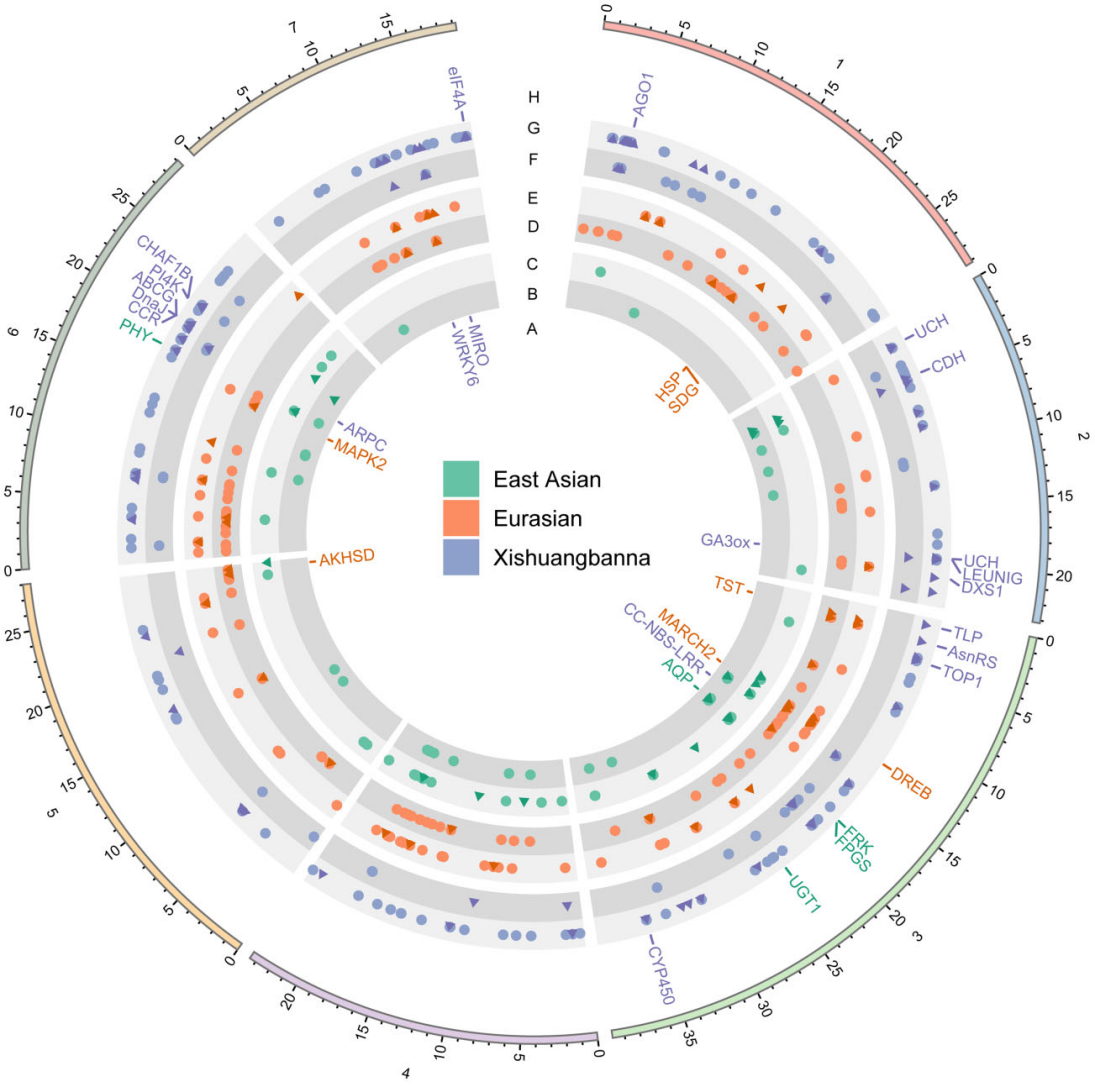
Genes harboring soft and hard selective sites in cultivated populations. Tracks indicate the following: genes containing only hard selective sites in the East Asian (green), Eurasian (orange), and Xishuangbanna (blue) populations (A); hard selective variants in the East Asian population (B); soft selective variants in the East Asian population (C); hard selective variants in the Eurasian population (D); soft selective variants in the Eurasian population (E); hard selective variants in the Xishuangbanna population (F); soft selective variants in the Xishuangbanna population (G); and genes containing only soft selective sites in East Asian (green), Eurasian (orange), and Xishuangbanna (blue) populations (H). In the B–F tracks, dark triangle dots indicate selective sites located in gene regions, and light circle dots indicate selective sites located in intergenic regions. The plot was generated using Circos v0.69-9 (Krzywinski *et al.* 2009).

The genes containing hard or soft selection sweep signatures (Fig. 4) were involved in developmental regulation, biotic, and abiotic stress resistance (Table 4). However, in the certain population, there were some differences. For example, for hard sweeps, signatures in East Asian were not related to biotic stress resistance and signatures in Xishuangbanna were not related to abiotic stress resistance; for soft sweeps, selective sites in Eurasian were not involved in abiotic stress resistance.

**Table 4.** Functions of genes capturing hard sweep and soft sweep signatures in the cultivated populations.

Population	Developmental regulation		Biotic stress resistance		Abiotic stress resistance	
	Hard sweeps	Soft sweeps	Hard sweeps	Soft sweeps	Hard sweeps	Soft sweeps
East Asian	+	+	−	+	+	+
Eurasian	+	+	+	+	+	−
Xishuangbanna	+	+	+	+	−	+

“+” was used to represent genes containing the sweep signatures involved in the corresponding function in the population, and “−” was used for the reverse.

## Discussion

In this study, we performed a comprehensive genome scan of genetic variation in cultivated cucumbers worldwide, attempting to find signals of population-specific selection in each cultivated population. Therefore, 4 haplotype-based approaches, iHS, nSL, XP-EHH, and Rsb, were chosen, and Fisher’s method was used to combine separate *P* values of the 4 methods to increase the power to pinpoint the selected sites (Alexandra *et al.* 2015). We finally identified 360 variants distributed in 22 genes in the East Asian populations, 2,248 variants located in 60 genes in the Eurasian population, and 3,867 variants in 120 genes in the Xishuangbanna population. We further surveyed uniquely selective sites in a certain population. We observed that genes involved in population-specific adaptation were related to the response to abiotic and biotic stress, developmental processes, and traits of fruit in the 3 cultivated populations. In addition, soft sweeps were found common in adaptation, and hard sweep and soft sweep signatures pointed to similar adaptions.

We combined the *P* values of the 4 methods to reduce false positivity and bias in a certain method (Grossman *et al.* 2010). Referring to previous research results, the origins of domesticated cucumber were traced back to more than 2,500 years (Qi *et al.* 2013; Chomicki *et al.* 2020). Based on the knowledge of haplotype-based calculator fitting for the identification of completed or ongoing selection in genomes (Szpiech and Hernandez 2014), EHH-based methods were chosen. When the beneficial allele frequency was low (0.1–0.3), which represented an ongoing selection, iHS and nSL performed well (Fan *et al.* 2016). XP-EHH and Rsb were powerful for detecting the selection of high-frequency or fixed alleles in one population but not in another population (Sabeti *et al.* 2007; Tang *et al.* 2007). In addition, the iHS and the XP-EHH statistics were complementary to each other and the combination could increase the power of pinpointing the selected sites (Vitti *et al.* 2013).

Compared with a previous study (Qi *et al.* 2013), only 2, 9, and 7 domestication sweep regions contained the selective sites identified by our analysis in the East Asian, Eurasian, and Xishuangbanna populations, respectively. The different results may be caused by different computing strategies and methods. In the previous study, they combined the East Asian, Eurasian, and Xishuangbanna populations into a single cultivated population. Then, methods based on site frequency spectrum were used. Regions with significantly lower diversity in cultivated cucumbers compared to wild cucumbers (the top 5% of π_*wild*_/π_*cultivated*_ values) were detected. Meanwhile, regions with the top 5% of XP-CLR values were identified. These shared regions between the analysis of genetic diversity and XP-CLR analysis were considered to be selective sweeps. In our analysis, we scanned the whole genome for each cultivated population by performing 4 haplotype-based approaches, including iHS, nSL, XP-EHH, and Rsb. Finally, we used Fisher’s combination method to combine the *P* values of the 4 methods, which can greatly increase the power to pinpoint the selected regions (Zaykin *et al.* 2007). In addition, the selective signatures persist for only ∼0.01 *N*_e_ generations in the haplotype structure, but for an order of magnitude longer time in the site frequency spectrum (Booker *et al.* 2017); thus, we were more likely to detect relatively recent signatures compared with the previous study.

We identified selective signatures in cucumber cultivated worldwide and attempted to provide the insight into the footprints of natural selection and domestication. Most genes involved in local adaptation signatures were observed to related to the response of the environment. For example, population-specific, selective signatures were involved in light, temperature resistance, and reproduction in the East Asian population, in light, heat stress, pathogen and herbivore resistance, and DNA repair in the Eurasian population, and in various microbial pathogens, salt and drought resistance in the Xishuangbanna population. These functions embodied the local adaptation to latitudes, daylight, soil conditions, and abiotic and biotic environments. In addition, the selective footprints associated with domestication were observed. For instance, selective gene related to shorter fruit length was detected in Eurasian; gene controlling flesh thickness was observed in the Xishuangbanna population. With the improvement of the genome functional annotation, we will completely understand the selection during cucumber domestication.

Selective sites were divided into soft or hard sweeps based on Bayes factors proposed by Garud *et al.* (2021). In East Asian and Xishuangbanna, soft sweeps were common, consistent with previous studies in maize (Beissinger *et al.* 2014). On the contrary, hard sweeps were common in Eurasian. Cucumber is monoecious crop with separate male and female flowers on the same plant. It is highly dependent on insect-mediated pollination but few dependent on wind and self-pollination is inefficient (Lowenstein *et al.* 2012). These characteristics of pollination system increase genetic diversity (Fattorini and Glover 2020) and provide standing variants for soft sweeps. The population size at bottleneck in the Eurasian population was larger than those in East Asian and Xishuangbanna (Qi *et al.* 2013), implying that standing variation enabled the Eurasian population to rapidly adapt the environment (Chaturvedi *et al.* 2021); however, duration of bottleneck in Eurasian was longer than those in East Asian and Xishuangbanna (Qi *et al.* 2013). These hinted that standing variation might contribute little to adaption of the environment and the Eurasian population recovery (Orr and Unckless 2014; Schrider and Kern 2017). Therefore, the proportion of soft sweeps in the Eurasian population was lower than those of other 2 cultivated populations. Genes covering hard and soft sweep signatures were involved in pathogen resistance, response to the environment and developmental regulation.

In conclusion, we found population-specific, recent selective signatures in the 3 cultivated populations. These selective sites are involved in development, response, resistance, and biosynthesis. We further observed that soft sweeps were common for cultivated populations in East Asian and Xishuangbanna, but the opposite pattern was observed in Eurasian, where hard seeps were common. Our methods provide guidance for the analysis of population-specific positive selection. Our results provide a comprehensive dataset of population-specific, selective signatures in cultivated cucumber. Importantly, these findings will help to further improve the experimental understanding of the biological mechanisms of local adaptation and domestication.

## Data availability

Supplementary Figs. 1–9 and Supplementary Tables 1–11 are available at figshare https://doi.org/10.6084/m9.figshare.19322318. Code used to combine *P* values using Fisher's combination can be found at https://github.com/CMB-BNU/fisher_combination. Supplementary Fig. 1: Comparison of our work and previous (Qi *et al.* 2013) in the statistics calculation strategy between populations. Supplementary Fig. 2: Phylogenetic tree of 7 closely related species in Cucurbitaceae. Supplementary Fig. 3: Decay of linkage disequilibrium (LD) in 4 populations. Supplementary Fig. 4: Manhattan plots of *P* values of iHS (A), nSL (B), XP-EHH (C), and Rsb (D) in East Asian. Supplementary Fig. 5: Manhattan plots of *P* values of iHS (A), nSL (B), XP-EHH (C), and Rsb (D) in Eurasian. Supplementary Fig. 6: Manhattan plots of *P* values of iHS (A), nSL (B), XP-EHH (C) and Rsb (D) in Xishuangbanna. Supplementary Fig. 7: Venn diagram for selective sites in East Asian. Supplementary Fig. 8: Venn diagram for selective sites in Eurasian. Supplementary Fig. 9: Venn diagram for selective sites in Xishuangbanna. Supplementary Table 1: Individuals and SNPs kept after quality filtering. Supplementary Table 2: Decay of linkage disequilibrium (LD), measured by *r*^2^ values, in 4 populations. Supplementary Table 3: The parameters in BEAGLE5.0 used to calculate haplotypes in each population. Supplementary Table 4: Signatures showing selection detected by the 4 methods in the cultivated populations. Supplementary Table 5: *P* values of the tests for equality of the proportion of selective signatures detected by each 2 methods in each cultivated population. Supplementary Table 6: States of selective SNPs only detected in the East Asian population. Supplementary Table 7: Functions of genes containing selective SNPs in the East Asian population. Supplementary Table 8: States of selective SNPs only detected in the Eurasian population. Supplementary Table 9: Functions of genes containing selective SNPs in the Eurasian population. Supplementary Table 10: States of selective SNPs only detected in the Xishuangbanna population. Supplementary Table 11: Functions of genes containing selective SNPs in the Xishuangbanna population.
